# Estrogen treatment following severe burn injury reduces brain inflammation and apoptotic signaling

**DOI:** 10.1186/1742-2094-6-30

**Published:** 2009-10-22

**Authors:** Joshua W Gatson, David L Maass, James W Simpkins, Ahamed H Idris, Joseph P Minei, Jane G Wigginton

**Affiliations:** 1 Department of Surgery, University of Texas Southwestern Medical Center, Dallas, TX 75390, USA; 2 DFW Center for Resuscitation Research, Dallas, TX, USA; 3 Department of Pharmacology and Neuroscience, UNTHSC, Fort Worth, TX 76107, USA

## Abstract

**Background:**

Patients with severe burn injury experience a rapid elevation in multiple circulating pro-inflammatory cytokines, with the levels correlating with both injury severity and outcome. Accumulations of these cytokines in animal models have been observed in remote organs, however data are lacking regarding early brain cytokine levels following burn injury, and the effects of estradiol on these levels. Using an experimental animal model, we studied the acute effects of a full-thickness third degree burn on brain levels of TNF-α, IL-1β, and IL-6 and the protective effects of acute estrogen treatment on these levels. Additionally, the acute administration of estrogen on regulation of inflammatory and apoptotic events in the brain following severe burn injury were studied through measuring the levels of phospho-ERK, phospho-Akt, active caspase-3, and PARP cleavage in the placebo and estrogen treated groups.

**Methods:**

In this study, 149 adult Sprague-Dawley male rats received 3rd degree 40% total body surface area (TBSA) burns. Fifteen minutes following burn injury, the animals received a subcutaneous injection of either placebo (n = 72) or 17 beta-estradiol (n = 72). Brains were harvested at 0.5, 1, 2, 4, 6, 8, 12, 18, and 24 hours after injury from the control (n = 5), placebo (n = 8/time point), and estrogen treated animals (n = 8/time point). The brain cytokine levels were measured using the ELISA method. In addition, we assessed the levels of phosphorylated-ERK, phosphorylated-Akt, active caspase-3, and the levels of cleaved PARP at the 24 hour time-point using Western blot analysis.

**Results:**

In burned rats, 17 beta-estradiol significantly decreased the levels of brain tissue TNF-α (~25%), IL-1β (~60%), and IL-6 (~90%) when compared to the placebo group. In addition, we determined that in the estrogen-treated rats there was an increase in the levels of phospho-ERK (*p *< 0.01) and Akt (*p *< 0.05) at the 24 hour time-point, and that 17 beta-estradiol blocked the activation of caspase-3 (*p *< 0.01) and subsequent cleavage of PARP (*p *< 0.05).

**Conclusion:**

Following severe burn injury, estrogens decrease both brain inflammation and the activation of apoptosis, represented by an increase in the levels of phospho-Akt and inhibition of caspase-3 activation and PARP cleavage. Results from these studies will help further our understanding of how estrogens protect the brain following burn injury, and may provide a novel, safe, and effective clinical treatment to combat remote secondary burn injury in the brain and to preserve cognition.

## Background

Burn injuries are a significant cause of death and disability around the world. In the United States, there are greater than 40,000 hospital visits for burn injury annually. Between 1995 to 2005 (National Burn Repository), there were 126,000 admissions to hospitals for burn injury [1]. A significant incidence of multiple organ (liver, thymus, spleen, skeletal muscle, lung, heart, and brain) dysfunction and failure has been noted in both animals and humans, surviving the initial insult of severe burn injury. This failure of different organ systems is thought to be, at least in part, due to increased apoptotic cell death and enhanced production of inflammatory cytokines such as interleukin-6 (IL-6), tumor necrosis factor-alpha (TNF-α), interleukin 1beta (IL-1β), and prostaglandins [2-4]. For example, following burn injury there is a significant increase in DNA damage and fragmentation in cardiac myocytes [5]. Additionally, in the heart, thymus, and spleen there is an increase in the levels of active caspase 3, 6, 8, and 9, which results in increased apoptotic cell death after burn injury in rats and mice [6-9]. Following severe burn injury, there is also an increase in signaling pathways such as the p38 and Jnk MAPKs leading to an amplification in cytokine production and the initiation of apoptosis [5,7,9,10]. With respect to the brain, previous studies have demonstrated that the brain is one of the remote organs subject to injurious effects following severe burn injury [10-15]. For example, neuropsychological disorders have been documented in children following burn injury. In these children, chronic mental disability was correlated with the size of the burn injury [12]. Additionally, a retrospective clinical study found that a small percentage of burn patients also suffer from acute stroke [11]. In animal studies of burn injury, magnetic resonance imaging has identified marked changes in the brain up to 3 days post-burn injury, most notably brain swelling and lesions [14]. Over the initial 24 hour post-burn period, rats have been found to display decreased glucose utilization in the brain, however by week three the glucose utilization returned to baseline, indicating an acute dysregulation of glucose metabolism in the brain [16]. In behavioral studies such animals appear to have long-term cognitive deficits associated with a disruption in the blood-brain barrier [17-19], increased inflammation [13], and/or altered metabolism in the brain [10,16].

Estrogens have been shown to significantly decrease the levels of inflammatory cytokines and improve outcomes in animal models of burn injury. Some of estrogen's protective mechanisms of action include both maintaining the integrity of the mitochondria and decreasing the activity of a multitude of pro-apoptotic factors [20,21]. In previous animal models of severe burn injury, estrogen treatment resulted in a decrease in systemic and local production of pro-inflammatory cytokines [22-24]. In these studies, estrogen decreased the levels of oxidative stress, inflammatory cytokines (IL-6, TNF-α), NF kappa beta (NFκβ) signaling, and importantly maintained the histological architecture of organs such as the stomach and significantly improved survival [22,24-28]. Since estrogens protect the brain from oxidative insults and inflammation in other models of acute brain injury such as stroke [29-33], we hypothesize that, following severe burn injury, a single dose of 17 β-estradiol would decrease inflammation in the brain, which might ultimately protect the brain from the injurious effects of a severe remote burn.

## Methods

### Experimental model

A total of 149 Adult male Sprague-Dawley rats weighing 325 to 350 g were obtained from Harlan Laboratories (Houston, TX) and housed in a University of Texas Southwestern Medical Center (UT Southwestern) animal care facility. Commercial rat chow and tap water were available ad libitum, and rats were maintained in a constant temperature environment with a 12-h light/dark cycle. All experiments were performed under a protocol approved by the UT Southwestern Institutional Animal Care and Research Advisory Committee, and the work conformed to all guidelines outlined in the Guide for the Care and Use of Laboratory Animals published by the American Physiological Society.

### Burn injury and estrogen treatment

Rats were deeply anesthetized with isoflurane, secured in a constructed template device that exposed 40% of the total body surface area (TBSA) of the animal, and then administered a full thickness scald burn as previously described [34]. Briefly, the back and flank skin were shaved, and the animal was secured into a specially designed template. The skin that was exposed through the template was immersed in 100°C water for 10 seconds to produce a full thickness dermal burn over 40% of the TBSA. This burn technique produces complete destruction of the underlying nerve tissue, resulting in a burn injury that should not result in pain to the subject animal. After immersion, the rats were immediately dried, followed by standard fluid resuscitation based on the Parkland Burn Formula consisting of 4 ml/kg per percent burn of lactated Ringer (LR) solution intra-peritoneally (IP), with 1/2 of the calculated volume being given immediately after completion of the burn injury, and the other half given 8 hours post-burn. In addition to the LR, rats also received 0.1 mg/kg of IP Buprenorphine for pain control. Following fluid resuscitation, each animal was placed in an individual cage that rested on a heating pad. Burned rats did not display discomfort or pain, moved freely about the cage, and consumed food and water within 15 minutes after recovering from isoflurane anesthesia. Control animals (n = 5) were administered sham burns, which were treated in a manner identical to the burn treatment except these rats were exposed to room-temperature water and received no study drug. Fifteen minutes following true burn injury, the burned animals received either a subcutaneous dose (SQ) of 0.5 mg/kg of 17 β-estradiol (n = 72) or an equivalent volume of placebo (vehicle, which was corn oil) (n = 72) based on a computer-generated randomization schedule. This subcutaneous delivery of 17 β-estradiol has previously been shown to produce similar pharmacokinetics to intravenous dosing in other animal models, but with greater ease of delivery [35]. All investigators were blinded to the treatment group of the animals. At 0.5, 1, 2, 4, 6, 8, 12, 18, and 24 hours post-burn, eight animals from the placebo group and eight animals from the estrogen group were sacrificed at each time point, the brains were harvested, and total protein isolated. Control animals were sacrificed and harvested in the same manner at the 24 hour time point.

### ELISA

The TNF-α, IL-1β, and IL-6 cytokines were measured from the brain using the Enzyme Linked-Immuno-Sorbent Assay (ELISA; Invitrogen, Camirillo, CA). In brief, 50 μl of each sample and the biotinylated anti-TNF-α, IL-1β, and IL-6 was added in duplicate to the appropriate 96-well plate and incubated for approximately 2 hours at room-temperature. Following the 2 hour treatment, the plates were washed and 100 μl of the Streptavidin-HRP was added for 30 minutes at room-temperature. After the 30 minute incubation, 100 μl of the Stabilized Chromogen was added to each well and 100 μl of stop solution was added to stop the reaction. The absorbance was detected at 450 nm.

### Western blot analysis

The harvested rat brains were placed in 0.5 ml of lysis buffer in order to isolate the total protein (50 mM Tris, pH7.4; 150 mM NaCl; 10% glycerol; 1 mM EGTA; 1 mM Na3VO4; 5 mM ZnCl2; 100 mM NaF; 1% Triton X-100; 10 mg/mL of aprotinin; 1 mg/mL of leupeptin; and 1 mM phenylmethylsulfonyl fluoride), then homogenized and centrifuged for 10 minutes. Following centrifugation, the supernatant was collected and analyzed for protein concentration. Protein concentrations were determined using the Bio-Rad DC protein assay kit (based on the method of Lowry; [36]). Total protein (100 μg) was loaded onto a sodium dodecyl sulfate (SDS), 10% polyacrylamide gel (PAGE) and run at 100 volts for 1 hour. After electrophoresis, the protein was transferred to a polyvinylidene difluoride membrane (PVDF; 0.22 mm pore size, Bio-Rad, Hercules, CA) and blocked for 3 hrs in a 3% bovine serum albumin (BSA), 0.2% Tween-containing TBS (TBS-T) solution. The membrane was subsequently probed with the phospho-p44/42 Map Kinase (1:1000; Cell Signaling), phospho Akt (1:1000; Cell Signaling), total Erk1/2 (1:1000; Cell Signaling), active caspase 3 (1:500; Sigma Aldrich), or the poly (ADP-ribose) polymerase (PARP; 1:200, Sigma Aldrich) antibodies. Antibody binding to the membrane was detected using a secondary antibody (goat anti-rabbit) conjugated to horseradish peroxidase (1:20,000; Pierce, Rockford, IL.) and visualized with the aid of an imaging system, using enzyme-linked chemiluminescence (ECL; Amersham, Arlington Heights, IL). All blots were re-probed with anti- Erk1/2 antibodies to ensure equal loading. Densitometric analyses of the protein levels was performed to conduct statistical analyses.

### Measurement of plasma 17 β-estradiol levels

17 β-estradiol levels were measured from the plasma of the control group, the placebo group, and the estrogen group using the commercially available estradiol radioimmunoassay kit (DSL-440, Diagnostic Systems Laboratories, Webster, TX.). The assay was performed according to the manufacturer's protocol as previously published [35]. In brief, plasma samples (100 μl) were collected and measured in triplicate. The values were compared to the standard curve and the lowest levels of serum 17 β-estradiol was approximately 5 pg/ml.

### Statistics

Data obtained from no fewer than six independent animals were analyzed using the Student's *t*-test analysis. Groups were considered to be significantly different if *P *values < 0.05 (SPSS; Chicago, IL). The data are presented as a bar graph depicting the mean ± SEM, using the GraphPad Software (San Diego, CA).

## Results

### Treatment with 17 β-estradiol decreases burn-induced inflammation in the brain

**Figure 1:**
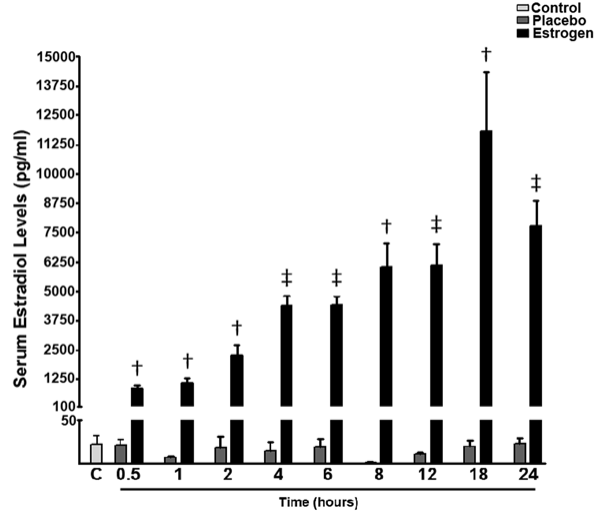
**Estrogen concentrations in rats following severe burn injury**. Using a radioimmunoassay method, we found that at 0.5, 1, 2, 4, 6, 8, 12, 18, and 24 hours post burn there was a significant increase in the levels of serum estradiol in those animals receiving a single dose of 17 β-estradiol. Depicted are mean ± SEM for n = 5. † *p *< 0.001, and ‡ *p *< 0.0001 vs. Placebo.

In the current study, our primary goal was to elucidate the effect of acute estrogen administration on the levels of inflammatory cytokines in the brain following burn injury. To evaluate the circulating 17 β-estradiol concentrations of all animals in the study, we measured the plasma 17 β-estradiol concentrations to determine the bioavailability of our steroid hormone preparation given SQ. We found that in those animals receiving SQ estradiol, there was a time-dependent increase in the levels of circulating 17 β-estradiol concentrations ranging from approximately 1 ng/ml to approximately 10 ng/ml. The basal levels of estradiol in both the control and placebo groups was approximately 20 pg/ml, and remained relatively constant at all measured time points (Fig. 1).

**Figure 2:**
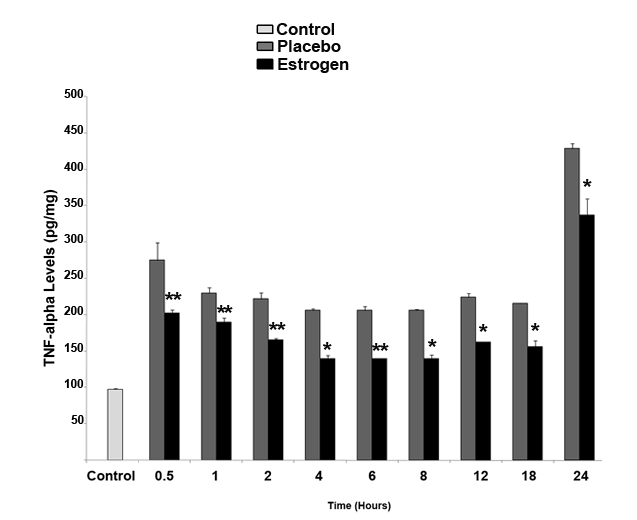
**Estrogen decreases the local concentration of TNF-α in the brain following severe burn injury**. Using the ELISA method, we found that at 0.5, 1, 2, 4, 6, 8, 12, 18, and 24 hours post burn there was a significant decrease in the levels of TNF-α in those animals receiving 17 β-estradiol compared with the placebo group. Depicted are mean ± SEM for n = 8. *p < 0.05, and **p < 0.01 vs. Placebo.

**Figure 3:**
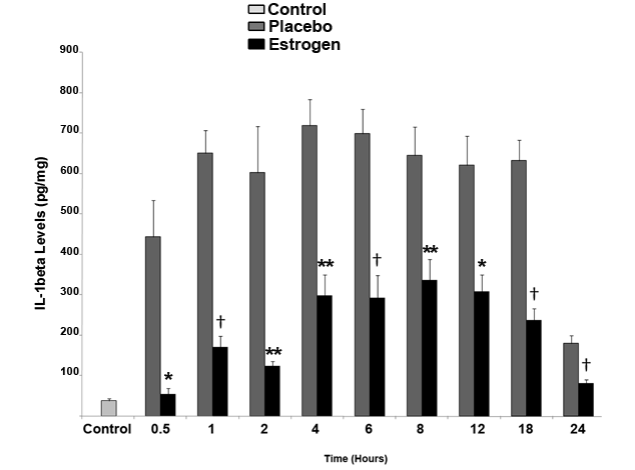
**After severe burn injury, estrogen significantly decreases the local levels of IL-1β in the brain**. At the 0.5, 1, 2, 4, 6, 8, 12, 18, and 24 hour time-points, there was a substantial decrease in the levels of IL-1β in the 17 β-estradiol treated group compared to placebo. Depicted are mean ± SEM for n = 8. *p < 0.05, **p < 0.01, † *p *< 0.001 vs. Placebo.

With respect to inflammatory cytokines measured in the rat brain tissue, at all evaluated time-points there was a significant reduction in TNF-α levels in the 17 β-estradiol group compared to the placebo group (*p < 0.05 at 4, 8, 12, 18, and 24 hours post-injury, and **p < 0.01 at 0.5, 1, 2, and 6 hours post-injury). Of interest, there was a near doubling in the levels of TNF-α in both the placebo and 17 β-estradiol groups noted at the 24 hour time-point compared to earlier time-points (Fig. 2). Analysis of IL-1β in the rat brain tissue revealed a 12-fold average increase in the IL-1β levels of the placebo-group compared to the control animals at all time points, however this difference waned significantly at twenty-four hours, where there was only a 3-fold increase in the levels of IL-1β in the placebo group compared with controls (Fig. 3). In the estrogen-treated animals, the levels of IL-1β were suppressed by approximately 50-85 percent compared with placebo-treated animals at all time points.

**Figure 4:**
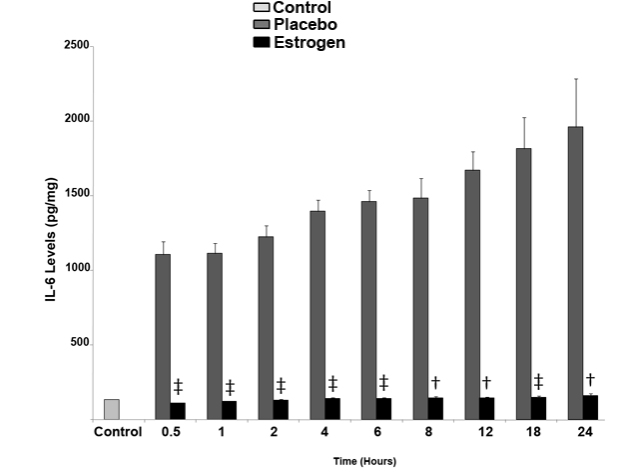
**Estrogen treatment results in a decrease in IL-6 levels in the brain following severe burn injury**. After burn injury and subsequent estrogen treatment, using the ELISA assay, we found that at 0.5, 1, 2, 4, 6, 8, 12, 18, and 24 hours post burn, there was a significant decrease (~90%) in the levels of IL-6 in animals receiving 17 β-estradiol compared with the placebo group. Depicted are mean ± SEM for n = 8. † *p *< 0.001, and ‡ *p *< 0.0001 vs. Placebo.

Most strikingly, the IL-6 cytokine levels in the brain tissue spiked rapidly, and continued to consistently increase over time at all measured intervals. In contrast, 17 β-estradiol treatment resulted in complete elimination of the burn-induced increase in IL-6 levels. Within the estrogen treated group, the levels of IL-6 were similar to the control group at all time points. Acute 17 β-estradiol treatment significantly lowered IL-6 levels in the brain tissue compared with the placebo-treated group (Fig. 4; †*p *< 0.001 at 8, 12, and 24 hours post-burn, and ‡p < 0.0001 at 0.5, 1, 2, 4, 6, and 18 hours post-burn).

### Estrogen increases ERK and Akt signaling in the brain following severe burn injury in rats

**Figure 5:**
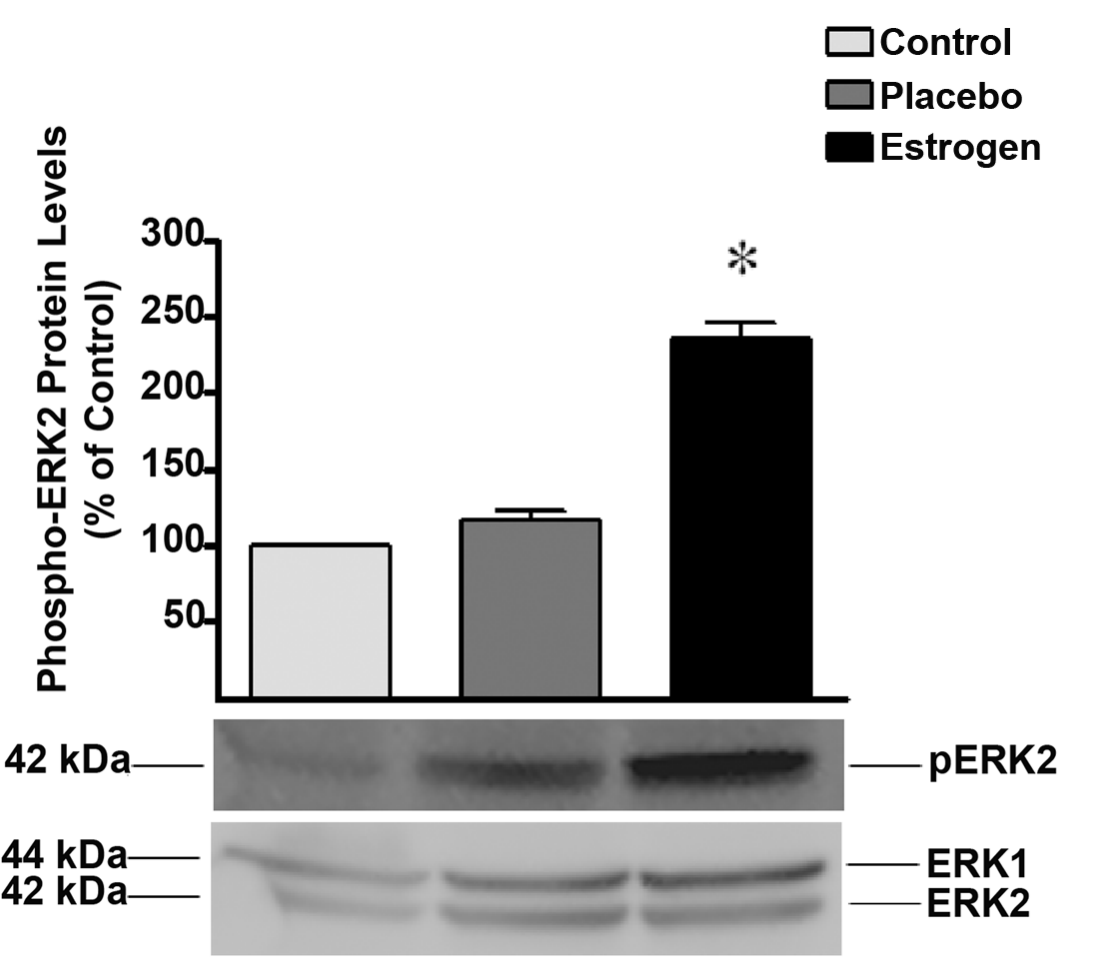
**Estrogen treatment results in a significant increase in the levels of phospho-ERK, following severe burn injury**. Using Western blot analysis, we found that there was a significant increase in the levels of phospho-ERK in the estrogen treated group at 24 hours post burn. The ERK1 and 2 blot displays equal loading between wells. Depicted are mean ± SEM for n = 5. *p < 0.01 vs. Placebo.

Since some of estrogen's protective effects are known to be mediated through ERK, we hypothesized that this steroid hormone would increase the activity of ERK in order to decrease inflammation and damage to the brain. In this study, we analyzed the levels of phospho-ERK using Western blot analysis to determine if estrogen modulates the activity of ERK in the brain following burn injury. We found that post-injury, estrogen treatment results in a 2.5-fold (*p *< 0.01) increase in the levels of phospho-ERK2 at the 24 hour time-point compared to the placebo and control groups (Fig. 5).

**Figure 6:**
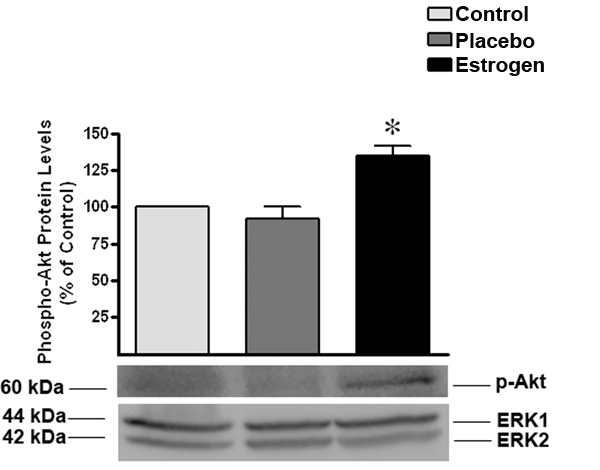
**Following burn injury, estrogen treatment results in a significant increase in the levels of phospho-Akt**. As indicated by Western blot analysis, we determined that there was a significant increase in the levels of phospho-Akt in the estrogen treated group at 24 hours post burn. The ERK1 and 2 blot displays equal loading between wells. Depicted are mean ± SEM for n = 6. *p < 0.05 vs. Placebo.

In prior burn studies, increased protein kinase B (Akt) signaling has resulted in a decrease in apoptosis in peripheral tissue, which has been identified as a major factor for cell survival following injury [37,38]. In the current study, we tested the hypothesis that estrogen increases the activity of Akt in the brain after burn injury and we found that estrogen significantly (*p *< 0.05) increased the levels of phospho-Akt by approximately 45 percent at 24 hours in the rat brain after severe burn injury. In the placebo group, there was no apparent increase in phospho-Akt levels compared to the control group (Fig. 6).

### Estrogen blocks caspase-3 activation and PARP inactivation after severe burn injury in rats

**Figure 7:**
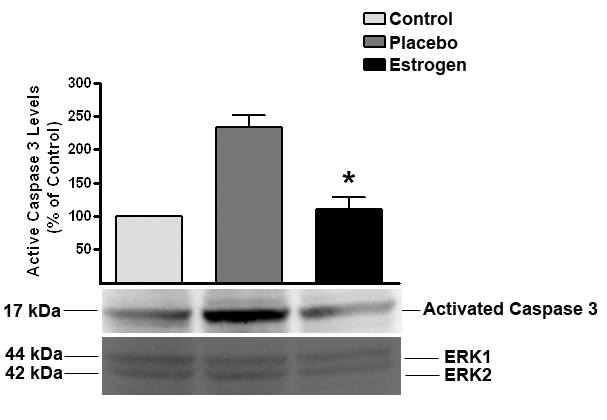
**Estrogen decreases the levels of activated caspase-3 after severe burn injury**. In these studies, we found that there was a significant decrease in the levels of activated caspase-3 in the estrogen treated group at 24 hours post burn. The ERK1 and 2 blot displays equal loading between wells. Depicted are mean ± SEM for n = 6. *p < 0.01 vs. Placebo.

To determine if estrogen blocks caspase activation in our burn model, we measured the levels of active caspase-3 in the 24 hour rat brain samples. We found that at 24 hours post-burn, the levels of active caspase-3 were unchanged in the estrogen group compared with the control animals, however there was a 2.5 fold increase in the levels of active caspase-3 in the placebo group compared with the estrogen group (*p *< 0.01) (Fig. 7).

**Figure 8:**
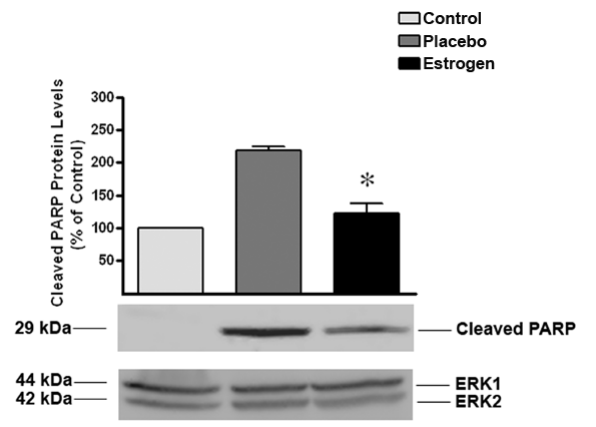
**Estrogen blocks burn-induced PARP inactivation**. Twenty four hours following burn injury and acute estrogen treatment, estrogen significantly blocked PARP cleavage. The ERK1 and 2 blot displays equal loading between wells. Depicted are mean ± SEM for n = 5. *p < 0.05 vs. Placebo.

To determine whether caspase-3 activation leads to increased apoptotic signaling in our burned animals, we measured the levels of cleaved Poly (ADP-ribose) Polymerase (PARP) from the brain tissues using Western blot analysis. PARP is a nuclear enzyme that recognizes and repairs damaged DNA. Following the initiation of apoptosis, the activated caspases cleave the PARP enzyme and block the repair of the DNA and cell survival [39-41]. The burned animals treated with estrogen exhibited low levels of cleaved PARP that were similar to the control group (*p *< 0.05), as opposed to a significant increase of cleaved PARP seen in those animals treated with placebo (Fig. 8).

## Discussion

Abnormally high levels of circulating and tissue cytokines, particularly IL-6, have been found to correlate with both morbidity and mortality in patients with severe burn injury [42-44]. In order to better explain these clinical observations, models of acute severe burn injury in animals have demonstrated that secondary injury in the periphery and central nervous system results in a disruption of both the metabolism and the activity of various signaling pathways [10]. This secondary burn injury consists, at least in part, of increased expression of pro-inflammatory cytokines and increased cytokine signaling [2-4]. Estrogen decreases this cytokine signaling by interacting with transcription factors in a receptor-dependent manner, and by decreasing the expression of cytokines such as IL-6, IL-1β, and TNF-α. Upon binding to either estrogen receptor (ER)-α or β, the resultant complex forms a homo-dimer that then binds to transcription factors at specific binding sequences. This binding blocks the ability of the transcription factors to initiate the transcription of numerous inflammatory cytokines [45-47]. In addition to the classical estrogen receptors, a membrane ER may also mediate the anti-inflammatory effects of estrogen following burn injury [48].

Estrogen's ability to decrease the levels of pro-inflammatory cytokines following severe burn injury may result in significant protection in the brain. Therefore, as a first investigative step, we chose to elucidate the early patterns of inflammation and apoptosis in the brain following severe burn injury, and to gauge the effects of estrogen on these changes. In this study, severe burn injury resulted in an extremely rapid and substantial increase in the levels of TNF-α, IL-1β, and IL-6 in the rat brain tissue (Fig. 2, 3, 4). We noted that in the estrogen treated group, the levels of these pro-inflammatory cytokines were markedly suppressed throughout all observed time-points, suggesting that an acute dose of estrogen effectively suppresses burn-induced surges in inflammatory cytokines in the brain. Secondarily, we evaluated the MAPK in the brains of these animals, to determine if the suppression of these cytokines by estrogen might be mediated by ERK. In other experimental models, estrogen administration has demonstrated modulation of the activity of signaling pathways such as the mitogen activated protein kinase (MAPK) within minutes of drug delivery. This effect of estrogen on intra-cellular signaling pathways is non-genomic, and leads to enhanced growth, cell survival, or even cell death, depending on the activation kinetics of the various signaling factors [49-57]. The activity of the ERK has been found to be increased in the brain at 6 hours following burn injury [5], demonstrating that following burn injury, there are significant changes in the MAPK signaling factors. Estrogen is known to activate the ERK pathway to promote cell survival or growth [30,54], and previous studies have shown that ERK blocks cytokine signaling [58,59]. Thus, the marked increase of phospho-ERK that we observed at the 24 hour time-point suggests that ERK may be an important mediator of estrogen's protective actions in the brain following severe burn injury. Based on our current findings, we believe that the estrogen-induced increase in ERK following burn injury results in a decrease in cytokine signaling and subsequent cytokine production. This decrease in inflammation could translate into less damage and cell loss in the brain, which may in the future be shown to preserve cognition in patients with severe burn injury.

This study could not determine if the observed profound increase in brain pro-inflammatory cytokines was due to local brain synthesis or from peripherally produced cytokines that penetrated into the brain. We believe that the observed burn-induced increase in pro-inflammatory cytokines was from local sources for two reasons. First, in this study, plasma levels of TNF-α, IL-1β and IL-6, while substantially increased following burn injury, were 10-fold lower than levels that we detected in the brain (Wigginton, JG *et al*., unpublished observation), suggesting that brain transport of these peripherally-derived cytokines against a concentration gradient is not likely. Second, glia and neurons are well known sources of brain cytokines and may be the source of local cytokine production in the brain following severe burn injury [60,61]. Our hypothesis that the elevated cytokine concentrations were produced in the brain is also supported by previous studies, which have demonstrated increases in stress in the brain as measured by electroencephalography following severe burn injury of peripheral tissue. This increased activity and brain inflammation is thought to be a result of increased nitric oxide signaling, resulting in increased production of inflammatory cytokines by astrocytes, microglia, and neurons [15,62]. Elevations of nitric oxide levels have been previously shown to increase inflammation and mortality following severe burn [63-65].

Because local accumulation of cytokines may induce apoptosis and significantly extend the initial injury, we also wanted to determine if estrogen's ability to block cytokine signaling would lead to a decrease in the activation of apoptotic proteins in the brain. In previous studies, severe burn injuries have been associated with a significant increase in apoptosis [5-9]. Here, we determined that estrogen administration following burn injury increased the activity of Akt and decreased the levels of active caspase-3. Akt is an anti-apoptotic factor that stabilizes the mitochondria. This mitochondrial stabilization is effected by decreasing cytochrome-C release (an initiator of apoptosis) and decreasing caspase activation [66-68]. As a result of these alterations, we noted a significant decrease in PARP cleavage, indicating that apoptotic episodes are decreased in the estrogen-treated groups. These studies support the idea that the brain undergoes early, marked changes following remote burn injury, which may be responsible for a dramatic increase in cell death and a subsequent reduction in brain function.

While these studies are extremely provocative, there are several limitations that should be addressed in future investigations. First, while both sexes of animals have been noted to benefit from early estrogen administration in many models of injury, including stroke and hemorrhagic shock [69,70], we chose to study intact male animals to reduce or eliminate the effects of endogenous estrogen and to focus solely on the effects of exogenous estrogen following burn injury. With regard to estrogen delivery, the subcutaneous route of administration in this study produced considerably elevated estradiol levels as early as 15 minutes post-administration, resulting in significant cytokine reductions at all time points. However, these elevations in circulating levels of estradiol were delayed compared with prior estradiol pharmacokinetic observations in the rodent model [35]. We hypothesize that the delay in the peak of systemic estradiol levels may be due to peripheral vasoconstriction following this large burn injury, and the clinical relevance of the severe burn injury model may be improved by intravenous administration of the drug. Additionally, we chose to build on our prior work by administering an extremely large burn to these animals. The effects on the brain and the effects of estrogen on smaller or larger injuries can only be hypothesized based on the current data. In addition, these animals were only allowed to survive for 24 hours following burn injury, as the nine time points observed in this study afforded us the opportunity to elucidate the rapid and dramatic changes in the brain following severe burn injury. While we structured this study to observe the pure, acute effects of estrogen administration on burn injury, it may be possible to improve the clinical significance of this animal model in future studies by increasing the survival time of the animals, and by also noting estrogen's effects on post-burn complications frequently seen in our clinical patients. Finally, while markers of inflammation and apoptosis are important and clinically relevant, future animal studies evaluating the changes in the rats' pre- and post-burn cognitive function could further solidify our current observations of estrogen's protective effects on the brain following burn injury.

Despite these limitations, we conclude that following severe burn injury in a rodent model, an early, single dose of estrogen dramatically reduces brain inflammation and apoptosis for up to 24 hours post-injury. While further studies are warranted, it is our hope that results from these studies will help guide the development of a safe, inexpensive, easy-to-deliver, and effective exciting new therapy aimed at significantly improving the outcomes of patients with severe burn injury in the near future.

**Figure 9:**
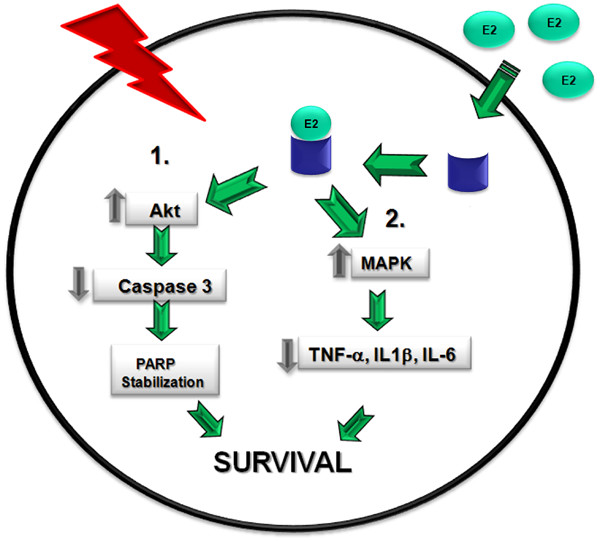
**Proposed model of estrogen's anti-inflammatory and pro-survival actions in the brain following severe burn injury**. In this study, we propose that following severe burn injury, acute estrogen treatment increases the activity of the anti-apoptotic Akt signaling protein and decreases the activity of pro-apoptotic factors such as caspase-3. Additionally, estrogen decreases PARP cleavage and ultimately decrease the levels of apoptosis (Pathway 1). Following injury, estrogen increases the activity of ERK through a parallel pathway, and subsequently decreases the levels of inflammatory cytokines (Pathway 2).

In conclusion, we observed that estrogen treatment protects the brain through two main pathways following severe burn injury in rats. In the first pathway, estrogen increases the levels of phospho-Akt, which leads to a decrease in caspase-3 activity and subsequent PARP cleavage, and ultimately protects the cell from increased programmed cell death. In a second pathway, we note that estrogen reduces inflammation in the brain, most likely by increasing ERK activation after burn injury. This increase in ERK activation by estrogen might lead to decreased cytokine signaling in cells and decreased brain inflammation (Fig. 9). These results support the conclusion that acute estrogen administration following severe burn injury in rats reduces early brain inflammation and apoptotic cell death.

## Competing interests

The authors declare that they have no competing interests.

## Authors' contributions

JWG participated in the design of the studies, personally conducted a significant portion of the experiments presented in the manuscript, and participated in the writing of the manuscript. DLM participated in the design and execution of the studies, and performed the ELISAs to detect the levels of the inflammatory cytokines. JWS participated in the experimental design, and in the editing of the manuscript. AHI assisted with the editing of the manuscript, as well as the statistical analyses of the reported data. JPM assisted with evaluation of the data and with the editing of the manuscript. JGW participated in the design of the experiments, funding of the projects, and preparation of the manuscript.
